# Anthropogenic Pb contribution in soils of Southeast China estimated by Pb isotopic ratios

**DOI:** 10.1038/s41598-020-79203-3

**Published:** 2020-12-17

**Authors:** Jianwu Li, Guoshuang Hao, Xudong Wang, Li Ruan, Jinjie Zhou

**Affiliations:** 1 Key Laboratory of Soil Contamination Bioremediation of Zhejiang Province, Zhejiang A & F University, Hangzhou, 311300 China; 2 National Center for Tea Improvement, Tea Research Institute, Chinese Academy of Agricultural Sciences, Hangzhou, 310008 China; 3 Jiande Agricultural Technology Extension Center, Hangzhou, 311600 China; 4 Soil Research Institute, Chinese Academy of Sciences, Nanjing, 210008 China; 5 People’s Government of Yan Tan Town, Wenzhou, 325100 China

**Keywords:** Environmental sciences, Element cycles

## Abstract

Isotopic ratios were used to identify the source of Lead (Pb) contamination in rural soils from Southeast China. Enrichment of Pb in surface soils was detected from three sampling locations, with the ^206^Pb/^207^Pb ratio indicating recent anthropogenic input. The ^206^Pb/^207^Pb ratio from deeper soil profiles reflected the ratio from parent basalt. Mass fractions of anthropogenic-derived Pb for soil samples in the upper profiles was as high as 50%, implying that surface soils in the current study were impacted by anthropogenic activity. The ^206^Pb/^207^Pb and ^208^Pb^/206^Pb ratios were similar to anthropogenic sources including the combustion of coal, which has been common practice in the region for 2500 years. Considering the relatively short history of petroleum use in this area and the rural location of soils, anthropogenic Pb source from coal burning was considered to be the main cause of lead pollution.

## Introduction

Heavy metals, such as Cu, Zn, Ni, Cd, Cr and Pb, can be major contaminants in the soil environment^1–7^. Lead (Pb) is one of the most widely studied metals in the soil environment due to its toxicity and widespread use^8,9^. Globally, soils receive and store anthropogenic Pb from sources including industrial wastes and emissions, motor vehicle emissions from Pb containing fuels and mining activities^10–13^. Pb is highly persistent in the environment and due to its toxicity, is of particular concern to human health^14,15^. Pb can be absorbed via ingestion of soil^16^ and water through inhalation of dust and dermal contact^17,18^, and consumption of vegetables grown in contaminated soil^19^. Pb biomagnifies through the food chain^16,18,20^, thus it is of concern in both developed and developing countries^14,20–22^. Soil Pb contamination, through the various exposure pathways, has been shown to result in elevated human blood Pb levels^23–25^. Literature supports the notion that children are more susceptible to elevated blood Pb concentrations, with inhibition of neurobehavioral performance, including a lower intelligence quotient (IQ), deficits in verbal memory and attention, learning failure and reading disabilities^26^. Due to the great toxicity of lead to the environment and ecology, many researchers have carried out studies of Pb pollution and remediation^27–29^.

Isotope methodologies have been increasingly applied to environmental studies of Pb contamination of air, soils, sediments and plants^30–33^. Pb in the environment has four isotopic forms, ^204^Pb, ^206^Pb, ^207^Pb and ^208^Pb^34^. The isotopic composition of Pb is fundamentally controlled by geological properties, and is not fractionated by weathering, transportation or biological processes. Thus, the use of Pb isotopic signatures can assist in the identification and quantification of Pb sources^35–38^. Therefore, assessing Pb isotopes allows us to understand anthropogenic lead pools and earth surface processes related to regolith development^39,40^.

While there is a sound body of research globally on the distribution of Pb in soil, the source of the Pb is not always well described. This is particularly the case in Southeast China where anthropogenic contributions to soil Pb content have not been thoroughly examined. As one of the well-developed regions of China, our study area has been undergoing rapid industrialization and urbanization, thus the need to better understand the risks of Pb in the soils, as well as understanding where the main sources of contamination arise^13,15,21,22,41^. The objective of this study was therefore to analyze Pb concentrations and isotopic compositions of three subtropical soils in Southeast China to examine the isotopic composition of Pb through the soil profile, identify likely sources for the contamination, and to calculate the relative contribution of natural and anthropogenic Pb sources.

## Results

### Properties of studied soil profiles

Physicochemical characteristics of the soils are shown in Table 1. The pH ranged from 5.77 to 6.42 (Table 1) and generally increased with depth across all 3 sites. The bulk density was lower in the A horizon (0.95–0.98 g cm^−3^) than in C horizon (1.03–1.09 g cm^−3^) for each soil profile. The soil organic matter shows a decreasing trend with depth, with maximum values up to 44.2 g kg^−1^, 35.4 g kg^−1^ and 32.3 g kg^−1^ in the A horizon of ZSJ, ZCR and ZAJ profiles, respectively. The ZSJ, ZCR and ZAJ soil profiles represented for Sanjie, Chongren and Anjishan of Zhejiang province, respectively (Table 1).

**Table 1:** Selected physicochemical properties of the studied soil profiles.

Profile	Location	Horizon	Depth (cm)	pH (H_2_O)	Dry bulk density (g cm^−3^)	SOM (g kg^−3^)
ZSJ	Sanjie, Shengzhou (29° 47′ N, 120° 51′ E)	A	0–10	5.99	0.98	44.2
		B	10–25	5.95	1.02	21.2
		BC	25–35	6.02	1.05	8.5
		C	35–65	6.39	1.07	4.3
ZCR	Chongren, Shengzhou (29° 39′ N, 120° 47′ E)	A	0–15	5.97	0.95	35.4
		B	15–65	5.86	0.99	19.8
		C	65–	6.42	1.03	2.9
ZAJ	Anjishan, Xinchang (29° 27′ N, 121° 02′ E)	A	0–10	5.83	0.98	32.3
		B	10–30	5.77	1.07	22.8
		C	30–65	6.23	1.09	5.9

### Lead elemental and isotopic geochemistry

Lead concentration of soils and basalt is shown in Table 2. Pb concentrations of soil samples were higher than the parent bedrock (2.2 mg kg^−1^). Pb concentrations were up to 17.3 mg kg^−1^, 15.6 mg kg^−1^ and 15.5 mg kg^−1^ of the A horizons for ZSJ, ZCR and ZAJ, respectively. Pb concentrations decreased with increasing soil depth. The results clearly demonstrate an enrichment of surface soil Pb concentrations.

**Table 2:** Lead concentrations and isotopic composition in soils.

Profiles	Horizon	Sample numbers	Pb (mg kg^−1^)	^206^Pb/^204^Pb	^207^Pb/^204^Pb	^208^Pb/^204^Pb	^208^Pb/^206^Pb	^206^Pb/^207^Pb
ZSJ	A	4	16.2 ± 0.5	18.342 ± 0.009	15.595 ± 0.008	38.503 ± 0.011	2.099 ± 0.006	1.176 ± 0.004
	B	3	8.9 ± 0.3	18.428 ± 0.008	15.624 ± 0.005	38.717 ± 0.015	2.101 ± 0.003	1.179 ± 0.002
	BC	2	5.4 ± 0.2	18.529 ± 0.007	15.630 ± 0.007	38.831 ± 0.009	2.096 ± 0.002	1.185 ± 0.002
	C	4	6.7 ± 0.2	18.392 ± 0.005	15.597 ± 0.009	38.640 ± 0.012	2.101 ± 0.005	1.179 ± 0.003
ZCR	A	4	15.1 ± 0.5	18.410 ± 0.006	15.610 ± 0.010	38.585 ± 0.010	2.096 ± 0.004	1.179 ± 0.004
	B	6	8.6 ± 0.4	18.517 ± 0.009	15.597 ± 0.006	38.698 ± 0.008	2.090 ± 0.002	1.187 ± 0.003
	C	5	2.5 ± 0.1	18.511 ± 0.008	15.611 ± 0.009	38.770 ± 0.011	2.094 ± 0.003	1.186 ± 0.001
ZAJ	A	4	15.2 ± 0.6	18.445 ± 0.006	15.680 ± 0.007	38.806 ± 0.009	2.104 ± 0.004	1.176 ± 0.002
	B	5	5.9 ± 0.2	18.515 ± 0.009	15.618 ± 0.009	38.768 ± 0.013	2.094 ± 0.006	1.185 ± 0.005
	C	4	3.2 ± 0.1	18.637 ± 0.008	15.718 ± 0.008	39.138 ± 0.008	2.100 ± 0.005	1.186 ± 0.002
Basalt	Parent rocks	3	2.2 ± 0.1	18.630 ± 0.007	15.572 ± 0.007	38.733 ± 0.009	2.079 ± 0.003	1.196 ± 0.001

For the deep soils, the ^206^Pb/^207^Pb ratios (Table 2) of the ZSJ, ZCR and ZAJ profiles (> 60 cm) are closer to basalt, implying an influence from the parent material with little anthropogenic Pb at depth. However, for the top soils, the Pb isotopic compositions were distinct from the parent material. The ^208^Pb^/206^Pb ratios of surface soil samples were higher than the parent material (2.079; Table 2). But the ^206^Pb/^207^Pb ratios of surface soil samples were much lower than the basalt (1.196) and increase with depth. The significantly low radiogenic ^206^Pb/^207^Pb ratio (1.175; n = 12) of the soils in the top 0–10 cm is close to anthropogenic Pb from fly ash in China^42^. Therefore evidence is provided here that the surface soils have been substantially influenced by anthropogenic Pb inputs.

## Discussion

### Characterizing anthropogenic Pb in soils

**Figure 1:**
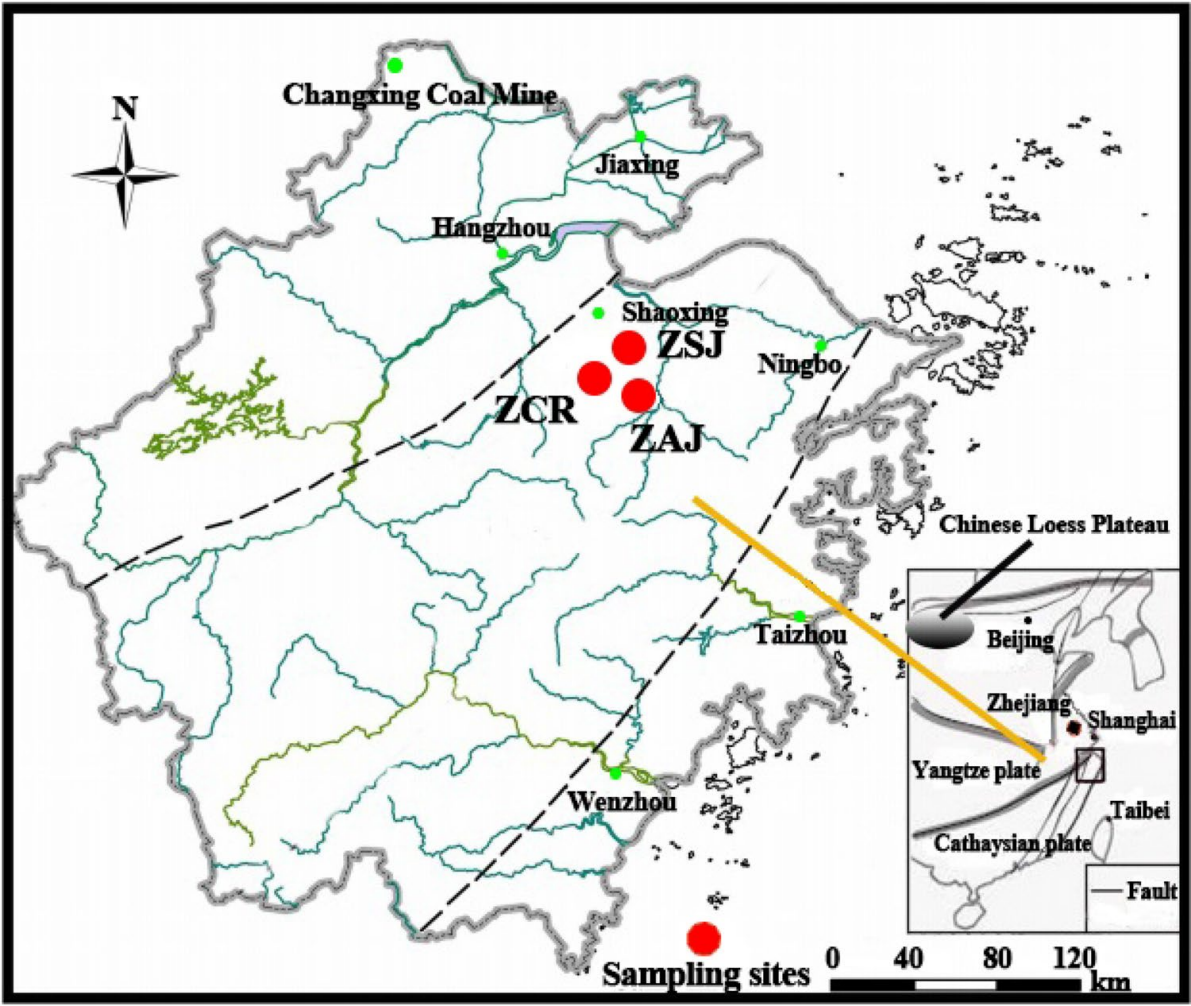
Pb content and ^206^Pb/^207^Pb ratios for soils in Southeast China.

**Figure 2:**
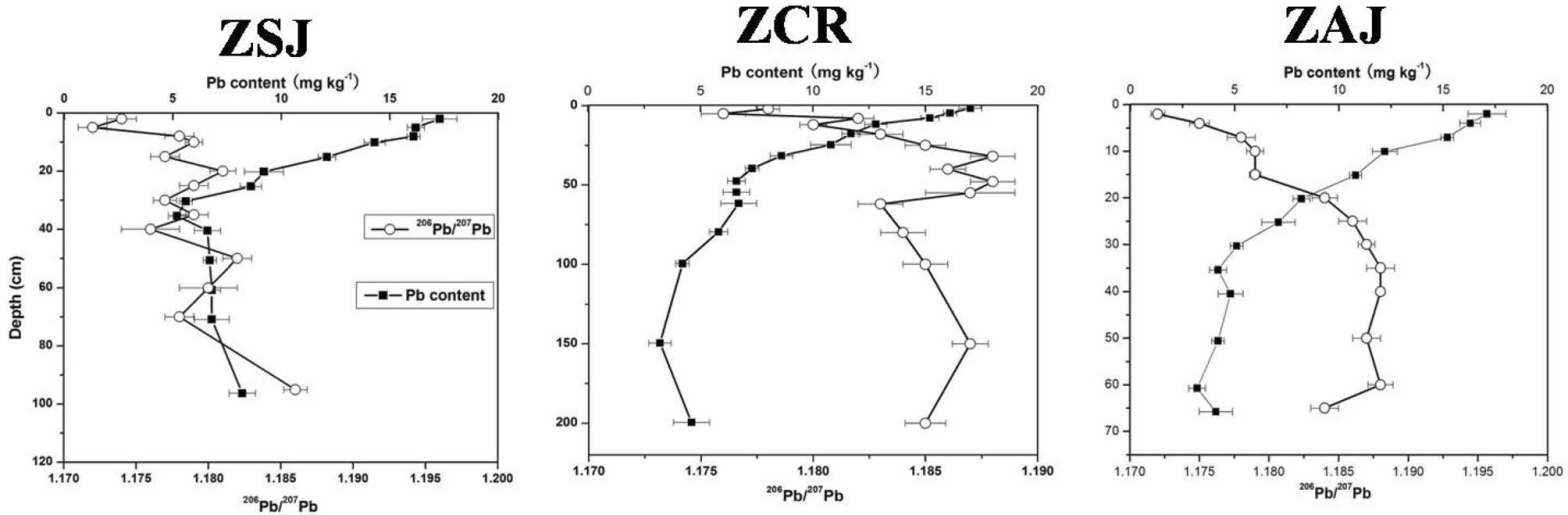
^208^Pb/^206^Pb vs. ^206^Pb/^207^Pb ratios. The ZSJ, ZCR and ZAJ soil profiles were represented for Sanjie, Chongren and Anjishan of Zhejiang province, respectively.

The ratio of ^206^Pb/^207^Pb was plotted against depth in comparison with Pb content (Fig. 1) illustrating that where higher Pb concentrations were detected (i.e. surface soils), there was a correspondingly lower ^206^Pb/^207^Pb ratio. The ^206^Pb/^207^Pb ratios decreased approximately with the increase of Pb concentration in soils (Fig. 1), suggesting an anthropogenic contribution to soil Pb concentrations. In order to help locate the source of Pb (i.e. naturally occurring from parent material, or anthropogenic), ^206^Pb/^207^Pb versus ^208^Pb^/206^Pb of soils, basalt and anthropogenic Pb sources were plotted (Fig. 2). The influence factors of human activities on Pb pollution mainly included smelting, automobile exhaust, coal combustion and so on. Firstly, the early Pb pollution was caused by emissions from the crude smelting technologies in copper production in Europe and China^43^. With the improvement of smelting technology and strict control of industrial pollution discharge, the contribution of smelting Pb to it is relatively small. Meanwhile, our research areas were remote from industrial areas, so smelting is not the main anthropogenic source of lead. Secondly, the anthropogenic Pb derived from the combustion of leaded petrol, often occurred in urban environments^44^, rather than in the rural areas. Our sample sites were far away from urban areas, so the effect of gasoline lead on it is relatively small. In addition, considering the shorter time usage of petroleum in China and the lower ^206^Pb/^207^Pb ratios for petroleum combustion (~ 1.11), its contribution to the change in soil Pb isotope ratios from ZSJ, ZCR and ZAJ could be considered as negligible^20,45,46^. Importantly, Pb ores from north China were different from the values of ZSJ, ZCR and ZAJ soils, with much higher ^208^Pb/^206^Pb ratios (2.15–2.33) and lower ^206^Pb/^207^Pb ratios (1.03–1.13)^47,48^. However, coal has been used in China for more than 2500 years. Coal combustion may be an important source of lead pollution in soil. The emission indicators of flue gas can be used to prove the conjecture of the source of Pb pollution. Recent studies have shown that the atmospheric lead emission from coal burning in China exceeded 10,000 t a^−1^ from 2001 to 2005, and the annual growth rate is 14.5%^49^. The highest average amount of lead discharged was in North China and the Shanxi, Shandong and Jiangsu province ranked the top three in terms of Pb discharge intensity. Lead emissions from these areas will be deposited in the study area along with the northeast monsoon^50^. Mukai et al.^48^ and Komárek et al.^51^ showed that the combustion of coal has an impact on aerosol Pb isotope ratios. The lower ^206^Pb/^207^Pb values in soil samples strongly indicate the coal combustion was the main cause of lead pollution in studied area. As shown in Fig. 2, the ^208^Pb^/206^Pb ratios are from 2.090 to 2.104, which were between basalt (2.079) and anthropogenic source from coal combustion (2.114); while the ^206^Pb/^207^Pb ratios range from 1.176 to 1.187, which are lower than their parent rocks (1.196) but higher than anthropogenic source from coal combustion in south China (1.162)^41^. After comprehensive consideration, we chose the average Pb isotope ratios of anthropogenic sources from coal combustion in Jiangsu-Zhejiang region to be ^206^Pb/^207^Pb = 1.162, and ^208^Pb/^206^Pb = 2.114.

### Calculation of anthropogenic Pb pools in soils of Southeast China

During thousands of years, different sources of anthropogenic Pb have been deposited on the surface of the soils. A two end-member model based on the isotope mass balance has been developed to calculate the percentage contribution of anthropogenic and natural Pb sources to total Pb in soils^13^. The Pb isotope ratio of basalts and anthropogenic source^41^ is ^206^Pb/^207^Pb = 1.196 and ^206^Pb/^207^Pb = 1.16, respectively.

1 $$f_{{{\text{anthropogenic}}}}^{Pb} = \frac{{R_{Pb}^{soil} - R_{Pb}^{basalt} }}{{R_{Pb}^{{{\text{anthropogenic}}}} - R_{Pb}^{basalt} }}$$

where $f_{{{\text{anthropogenic}}}}^{Pb}$ represented the percentage contribution of anthropogenic Pb source in soils, and the $R_{Pb}^{soil}$, $R_{Pb}^{{{\text{anthropogenic}}}}$ and $R_{Pb}^{basalt}$ are the Pb isotope ratios of soils, anthropogenic-derived and basalt-derived, respectively.

**Figure 3:**
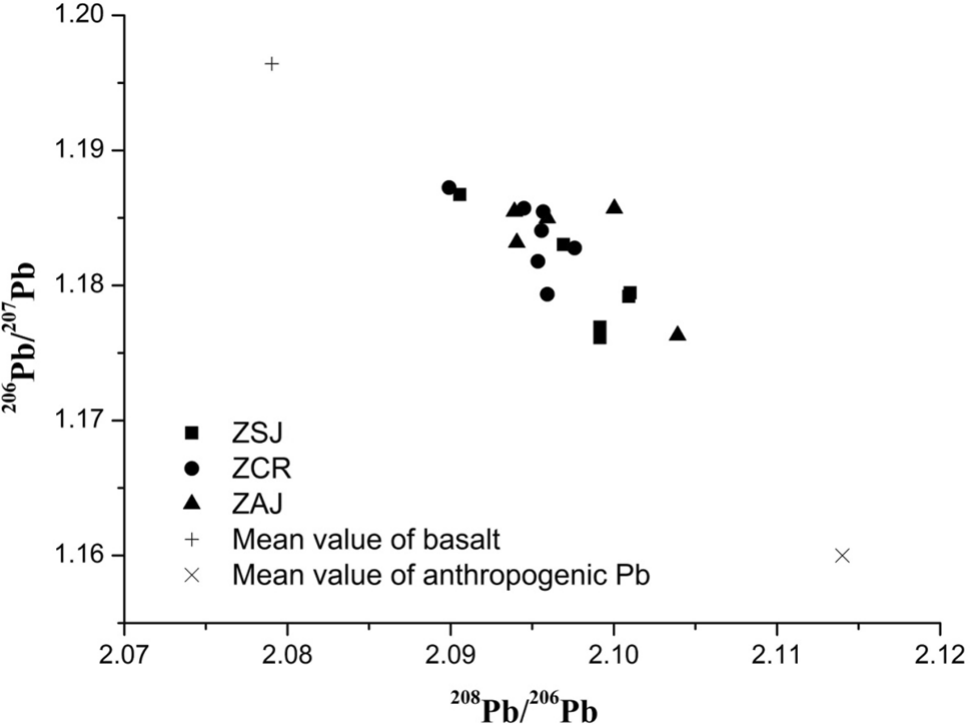
Mass fraction of anthropogenic Pb for soils in Southeast China.

Soils developed on the basalt from the study area are significantly influenced by contributions of anthropogenic Pb sources. The mass fraction (Fig. 3) of anthropogenic Pb ($f_{{{\text{anthropogenic}}}}^{Pb}$) from the ZSJ, ZCR and ZAJ profiles ranged from 25.78 to 55.20%, 24.33 to 46.26% and 29.24% to 54.71%, respectively. Moreover, the $f_{{{\text{anthropogenic}}}}^{Pb}$ values showed a prominent increase from the lower horizon to the surface horizon for all profiles tested. For the lower horizon (C horizon), the $f_{{{\text{anthropogenic}}}}^{Pb}$ values are lower, which indicates a primary influence from parent material. In contrast, for the topsoil (especially the A horizon), contributions of anthropogenic Pb were high (> 50%), implying large anthropogenic Pb addition to the soils in Southeast China.

Because the relatively short history of petroleum use in this area and the rural location of ZSJ, ZCR and ZAJ, with little vehicular access, local anthropogenic Pb source from gasoline are likely to have only a very minor influence on soil contamination. However, coal usage had long history in China. Ancient mining and utilization of coal were begun at Spring and Autumn and Warring States (470 B.C.), especially in the Sui and Tang Dynasties, the scale of coal mining and utilization was further expanded^52^. Large coal mines distribution include Hancheng (Shaanxi Province), Taiyuan and Changzhi (Shanxi Province), Yangzhou (Jiangsu Province) and Huainan and Huaibei (Anhui Province). In addition, as the largest coal mine in Zhejiang Province, Changxing coal mine is the nearest to the research area. In the northern winter season, cold air from high latitudes is controlled by the continental high-pressure system, and propagates southward to form the strongest northerly dry and cold winter monsoon in the world. The northern winter monsoon can controls the atmospheric circulation^50^ and carry the Pb pollutants from above coal mines to the study area during the dry season from November to April^53^. Meanwhile, Pb isotope ratios of the soils in this area were similar to that of anthropogenic Pb from coal combustion in China, particularly that of Jiangsu-Zhejiang region^41^, which is neighboring with Xinchang-Shengzhou Basin. Thus, we conclude that coal combustion is the main factor for the enhanced Pb contamination in surface soils.

In conclusion, three soil profiles from rural Southeast China been shown to have elevated surface Pb contamination. Using isotopic methodologies, this elevated Pb was shown to result mainly from anthropogenic activity. The ^206^Pb/^207^Pb values of deep horizons were close to the parent material suggesting contamination was restricted to the surface soil and did not leach through the profile. Our study suggested that the combustion of coal was the main source of soil contamination, and to avoid future contamination, lower particulate emissions will be required to avoid continued accumulation of Pb in surface soils in the region.

## Methods

### Study region and soil sampling

**Figure 4:**
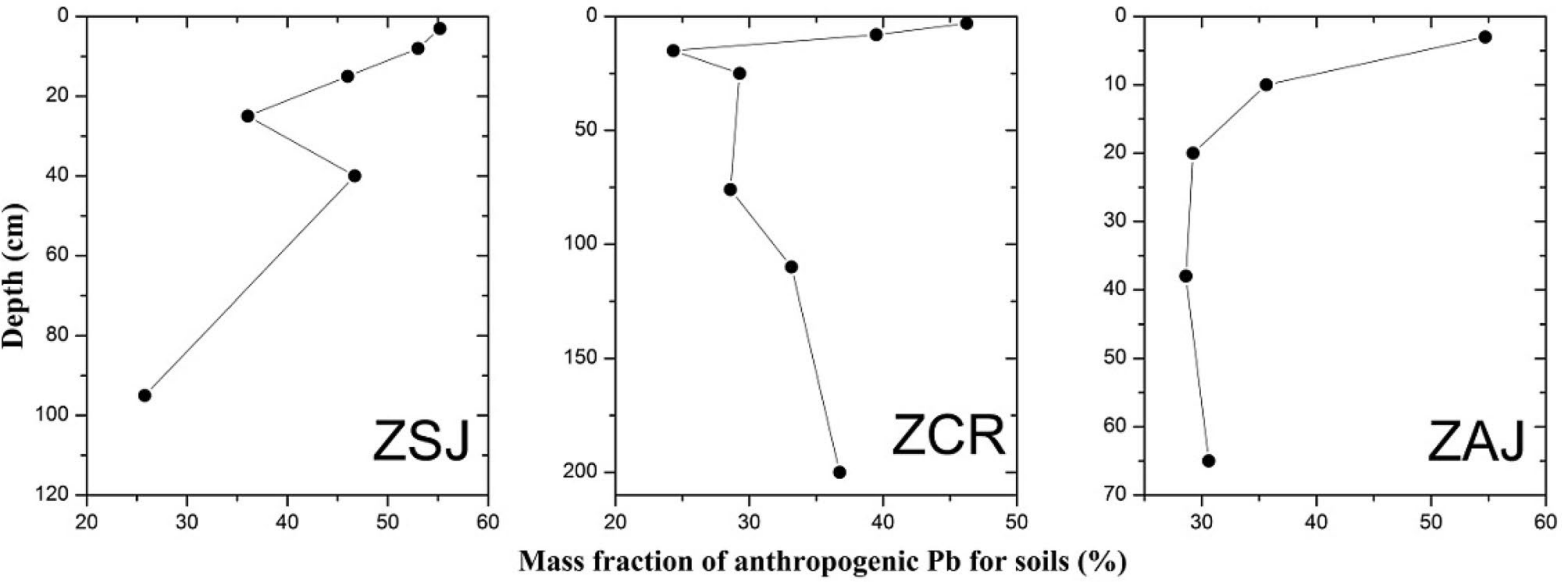
The location of sampling sites.

The study area is located in Xinchang-Shengzhou Basin, Southeast China, between 120° 2′ E–121° 0′ E and 29° 1′ N–29° 5′ N (Fig. 4). It belongs to the southern fringe of the northern subtropics^54^ and has a mean annual air temperature of 16.6 °C, with yearly extremes ranging from − 5.3 to 40.3 °C. The region has a mean annual precipitation of 1500 mm with nearly 70% falling during the wet season (April–September). Basalt is the dominant bedrock in the region^55^ with the resulting soil most commonly derived from in situ weathering of basalt. The soil is classified as either Udic Ferrosols^56^, or Ultisol according to USDA Soil Taxonomy^57^. The soils support plants that are dominated by *Machilus thunbergii* and *Camellia* sp. Three basaltic weathering profiles i.e. native forest soils (ZCR and ZAJ) and farmland soil (ZSJ), were selected in a rural area of Chongren, Anjishan and Sanjie respectively, in Zhejiang province (Fig. 4, Table 1), with locations being relatively remote from cities and obvious influences of human activity. The typical basalt platforms in the study area are distributed in triangles. We chose the north, southeast and southwest of the triangle platform as the sampling sites, in order to make the sampling points have better typical representative. The parent rock from all profiles was fresh tholeiitic basalt, which was collected beneath the sampling profiles. Soils were excavated to bedrock and sampled from small concavities in an otherwise convex portion of the landscape by genetic horizon.

### Laboratory analytical methods

Collected soil samples were air-dried, ground and passed through a 2 mm sieve. The soil pH was determined in a suspension of 1:2.5 soil:water solution (w/v). Soil bulk density was measured from the 100 cm^−3^ undisturbed soil cores by drying the cores for 24 h at 105 °C. A homogenized subsample of soil was digested with an acid solution (5 ml concentrated HNO_3_ (65%, v/v), 5 ml concentrated HCl (30%, v/v) and 5 ml concentrated HF (40%, v/v)). Diluted and filtered samples were assayed using an inductively coupled plasma mass spectrometer (ICP-MS) at the State Key Laboratory of Ore Deposit Geochemistry, Institute of Geochemistry in the Chinese Academy of Science^58^. The standard reference materials were GSR-3, BCR-1, GXR-5 and GXR-6. Analytical uncertainties were less than ± 5%.

For the determination of Pb isotopes, soil samples (0.05 g) were digested in a mixture of 4 ml concentrated HNO_3_ (65%, v/v) and 1 ml concentrated HF (40%, v/v) in Teflon vessels on a hotplate at 200 °C for 8 h. The vessel was then uncovered to allow evaporation to almost dryness. This procedure was repeated until the samples were completely dissolved^59^. Pb isotopes were measured on a GV Isoprobe-T thermal ionization mass spectrometer (TIMS) at the University of Science and Technology of China. The reagent blank was also measured and blank subtraction was done for the final intensity of each isotope of Pb in the sample. The relative standard deviations (RSD) of 10 replicate readings of samples were better than 1% for ^206^Pb/^207^Pb and 0.6% for ^208^Pb/^206^Pb. The average of measured ^206^Pb/^207^Pb and ^208^Pb/^206^Pb of the National Institute of Standards and Technology (NIST 981) were 0.9147 ± 0.0084 and 2.1681 ± 0.0099 with the certified values of 0.9147 and 2.1683, respectively.
