# Publisher Correction: Anthropogenic Pb contribution in soils of Southeast China estimated by Pb isotopic ratios

**DOI:** 10.1038/s41598-021-83013-6

**Published:** 2021-02-08

**Authors:** Jianwu Li, Guoshuang Hao, Xudong Wang, Li Ruan, Jinjie Zhou

**Affiliations:** 1 Key Laboratory of Soil Contamination Bioremediation of Zhejiang Province, Zhejiang A & F University, Hangzhou, 311300 China; 2 National Center for Tea Improvement, Tea Research Institute, Chinese Academy of Agricultural Sciences, Hangzhou, 310008 China; 3 Jiande Agricultural Technology Extension Center, Hangzhou, 311600 China; 4 Soil Research Institute, Chinese Academy of Sciences, Nanjing, 210008 China; 5 People’s Government of Yan Tan Town, Wenzhou, 325100 China

Correction to: *Scientific Reports* 10.1038/s41598-020-79203-3, published online 17 December 2020

This Article contains an error in the Figure legends of Figure 1, 2, 3 and 4. The legends of these Figures were inadvertently switched.

The legend of Figure 1:

“Pb content and ^206^Pb/^207^Pb ratios for soils in Southeast China.”

should read:

“The location of sampling sites.”

The legend of Figure 2:

“^208^Pb/^206^Pb vs. ^206^Pb/^207^Pb ratios. The ZSJ, ZCR and ZAJ soil profiles were represented for Sanjie, Chongren and Anjishan of Zhejiang province, respectively.”

should read:

“Pb content and ^206^Pb/^207^Pb ratios for soils in Southeast China.”

The legend of Figure 3:

“Mass fraction of anthropogenic Pb for soils in Southeast China.”

should read:

“^208^Pb/^206^Pb vs. ^206^Pb/^207^Pb ratios. The ZSJ, ZCR and ZAJ soil profiles were represented for Sanjie, Chongren and Anjishan of Zhejiang province, respectively.”

The legend of Figure 4:

“The location of sampling sites.”

should read:

“Mass fraction of anthropogenic Pb for soils in Southeast China.”

